# PET/MRI and PET/CT hybrid imaging of rectal cancer – description and initial observations from the RECTOPET (REctal Cancer trial on PET/MRI/CT) study

**DOI:** 10.1186/s40644-019-0237-1

**Published:** 2019-07-23

**Authors:** Miriam K. Rutegård, Malin Båtsman, Jan Axelsson, Patrik Brynolfsson, Fredrik Brännström, Jörgen Rutegård, Ingrid Ljuslinder, Lennart Blomqvist, Richard Palmqvist, Martin Rutegård, Katrine Riklund

**Affiliations:** 1 Department of Radiation Sciences, Diagnostic Radiology, Umeå University, SE-901 85 Umeå, Sweden; 2 Department of Medical Biosciences, Pathology, Umeå University, Umeå, Sweden; 3 Department of Surgical and Perioperative Sciences, Surgery, Umeå University, Umeå, Sweden; 4 Department of Radiation Sciences, Oncology, Umeå University, Umeå, Sweden; 5 Department of Molecular Medicine and Surgery, Karolinska Institutet, Stockholm, Sweden; 6 Department of Imaging and Physiology, Karolinska University Hospital, Solna, Sweden; 7 Wallenberg Centre for Molecular Medicine, Umeå University, Umeå, Sweden

**Keywords:** Rectal neoplasm, Rectal tumour, Staging, Lymph nodes, Tumour deposits, PET/CT, FDG-PET/CT, PET/MRI, FDG-PET/MRI

## Abstract

**Purpose:**

The role of hybrid imaging using ^18^F-fluoro-2-deoxy-D-glucose positron-emission tomography (FDG-PET), computed tomography (CT) and magnetic resonance imaging (MRI) to improve preoperative evaluation of rectal cancer is largely unknown. To investigate this, the RECTOPET (REctal Cancer Trial on PET/MRI/CT) study has been launched with the aim to assess staging and restaging of primary rectal cancer. This report presents the study workflow and the initial experiences of the impact of PET/CT on staging and management of the first patients included in the RECTOPET study.

**Methods:**

This prospective cohort study, initiated in September 2016, is actively recruiting patients from Region Västerbotten in Sweden. This pilot study includes patients recruited and followed up until December 2017. All patients had a biopsy-verified rectal adenocarcinoma and underwent a minimum of one preoperative FDG-PET/CT and FDG-PET/MRI examination. These patients were referred to the colorectal cancer multidisciplinary team meeting at Umeå University Hospital. All available data were evaluated when making management recommendations. The clinical course was noted and changes consequent to PET imaging were described; surgical specimens underwent dedicated MRI for anatomical matching between imaging and histopathology.

**Results:**

Twenty-four patients have so far been included in the study. Four patients were deemed unresectable, while 19 patients underwent or were scheduled for surgery; one patient was enrolled in a watch-and-wait programme after restaging. Consequent to taking part in the study, two patients were upstaged to M1 disease: one patient was diagnosed with a solitary hepatic metastasis detected using PET/CT and underwent metastasectomy prior to rectal cancer surgery, while one patient with a small, but metabolically active, lung nodulus experienced no change of management. PET/MRI did not contribute to any recorded change in patient management.

**Conclusions:**

The RECTOPET study investigating the role of PET/CT and PET/MRI for preoperative staging of primary rectal cancer patients will provide novel data that clarify the value of adding hybrid to conventional imaging, and the role of PET/CT versus PET/MRI.

**Trial registration:**

NCT03846882.

## Introduction

Imaging is of paramount importance when tailoring treatment strategies in patients with rectal cancer. Together with clinical examination and endoscopy, magnetic resonance imaging (MRI) of the pelvis is essential both in the initial setting and in restaging [1–3], providing assessment of the four main risk factors for disease recurrence: the extent of the primary tumour (T stage); metastases to locoregional lymph nodes (N stage); the presence of extramural venous invasion (EMVI); and the distance from the primary tumour to the mesorectal fascia (MRF). Of these risk factors, the N status is the most challenging to evaluate [4–8]. Furthermore, it is difficult to distinguish lymph nodes from tumour deposits. This is clinically important as recent studies have suggested that tumour deposits are associated with a worse survival outcome than metastatic lymph nodes alone [9–11]. In patients with a clinical complete response or near complete response after neoadjuvant therapy (short-term radiotherapy [RT], chemoradiotherapy [CRT] and chemotherapy), organ-saving strategies such as local excision and the watch-and-wait approach are now also valid options [2, 12], highlighting further the importance of accurate restaging after neoadjuvant treatment.

For primary assessment of possible metastatic disease (M stage), the standard procedure is computed tomography (CT) of the thorax and abdomen. Other modalities, such as positron emission tomography using ^18^F-fluoro-2-deoxy-D-glucose (FDG-PET) combined with CT, i.e. FDG-PET/CT, are considered in selected cases, but this is not recommended for evaluating local spread. It is mainly used in cases of equivocal distant spread and/or recurrent disease [13, 14].

3 T MRI scanners with integrated PET have become increasingly available at larger medical centres, and integrated PET/MRI is already performed in clinical practice as an alternative to PET/CT when optimal soft-tissue contrast is of importance, i.e. in head-and-neck, brain, solid organ, and musculoskeletal imaging, and when imaging children and women of fertile age [15]. Although there are a number of studies evaluating the role of integrated PET/MR imaging in colorectal patients [16–20], dedicated assessment of locoregional tumour involvement in primary rectal cancer has hitherto been evaluated sparsely, though with promising initial results [17].

Hypothetically, integrated FDG-PET/MRI could improve staging and restaging of rectal cancer either as a single imaging method or in addition to the routine clinical workup. This would be particularly advantageous in extramural disease, whether in regard to N status, EMVI, tumour deposits or MRF involvement. The ongoing prospective RECTOPET (REctal Cancer Trial On PET/MRI/CT) study primarily aims to evaluate PET/MR imaging in the pelvis in addition to conventional diagnostic MRI for locoregional staging/restaging of rectal cancer, while the secondary aim is to evaluate the role of PET/CT in this patient group. The aim of the current report is to present a feasible workflow for including PET/CT and PET/MRI in the clinical practice as well as the first experiences of the impact of PET/CT on staging and treatment for the patients included in the RECTOPET study.

## Materials and methods

### Patient population

All patients in Region Västerbotten, Sweden, in whom rectal cancer was strongly suspected, were eligible for inclusion in this prospective observational cohort study (NCT03846882). Patients were enrolled by surgeons and gastroenterologists from all three hospitals in the catchment area. Inclusion started in September 2016 and recruitment is still ongoing. The study was approved by the Regional Ethical Review Board in Umeå, by the Radiation Protection Committee in Umeå, and participation required written informed consent from each patient.

The inclusion criteria were: a) clinical- and/or biopsy-proven diagnosis of rectal cancer (inferior tumour margin less than 15 cm from the anal verge), b) written informed consent, and c) age over 18 years. The exclusion criteria were: a) impaired renal function, b) any contraindication to iodinated contrast media, c) contraindications for MRI, d) pregnancy and breastfeeding, e) a diagnosis other than rectal cancer, verified at biopsy e.g. benign dysplasia and anal cancer, and f) emergency surgery.

**Fig. 1:**
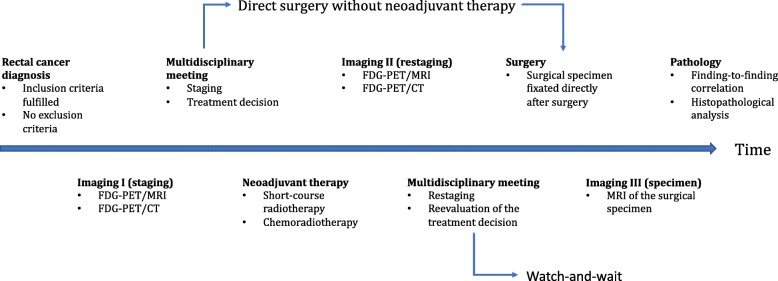
Flowchart for the included patients in the RECTOPET study

All the included patients were examined preoperatively with FDG-PET/CT and FDG-PET/MRI, replacing the conventional CT and MRI examinations performed as part of the clinical routine. For patients receiving neoadjuvant treatment followed by delayed surgery, a restaging second FDG-PET/CT and FDG-PET/MRI was performed preoperatively, 6 to 8 weeks after completed neoadjuvant treatment. Postoperatively, MRI of the surgical specimen was performed and, finally, histopathological analysis with finding-to-finding anatomical matching was performed. The flowchart in Fig. 1 presents the path of the included patients through the various examinations involved in the RECTOPET study.

### Patient characteristics

By December 2017 a total of 27 patients had been included. Three patients were subsequently excluded: one due to a histopathologically verified anal cancer; one due to an adenoma with low grade dysplasia; and one because the patient decided to abandon the MRI examination due to the noise. This gave a total study population of 24 patients (13 men, 11 women; median age 71 years, range 45–91) of which four had unresectable disease and were treated with palliative radiation and/or chemotherapy. Of the remaining 20 patients, 14 had elective surgery (13 with abdominal resection and one with transanal endoscopic microsurgery), one patient with a clinical complete response after neoadjuvant treatment was followed up in a watch-and-wait programme, and five patients were scheduled for surgery. The 14 patients who had undergone surgery provided 13 surgical specimens for MRI examination. Clinical details are presented in Table 1.

**Table 1:** Clinical and imaging data for the first 24 patients included in the RECTOPET study

Variables	N	%
Sex		
Male	13	54
Female	11	46
Tumour height		
≤ 5 cm	11	46
6–10 cm	8	33
11–15 cm	5	21
cT stage		
0	1	4
1	1	4
2	8	33
3	12	50
4	2	8
cN stage		
0	15	63
1	3	13
2	6	25
cM stage		
0	14	58
1	9	38
X	1	4
Management		
Direct surgery	5	21
Radiotherapy^a^	10	42
Chemoradiotherapy	3	13
Palliative	4	17
Other^b^	2	8
pT stage		
0^c^	3	0
1	2	8
2	3	13
3	10	42
4	0	0
No specimen yet/ever	6	25
pN stage		
0	10	42
1	6	25
2	2	8
No specimen yet/ever	6	25
Adjuvant therapy		
No	5	25
Yes	5	46

*c* clinical, *p* pathological

^a^One patient with a clinical complete response, in watch-and-wait programme; one patient declined curative surgery due to intervening femur fracture

^b^One patient referred for surgery of a synchronous lung metastasis from renal carcinoma; one patient underwent diagnostic transanal endoscopic microsurgery

^c^One patient with dysplasia; one patient with complete pathological response; one patient with no residual tumour

### FDG-PET/CT

The patients fasted for at least 6 h prior to the PET/CT, and a pre-examination blood glucose value below 11 mmol/L was verified in accordance with the European Association of Nuclear Medicine guidelines [21]. Image acquisition was performed using a Discovery 690 PET/CT scanner (GE Healthcare, Milwaukee, WI, USA), in supine position with arms raised above the head, from the orbitomeatal plane to the proximal thighs. Initially, a low-dose 120 kV 30 mA CT was acquired for attenuation correction. PET acquisition commenced 60 min post-injection of 4 MBq/kg, using a 2-min per bed position acquisition in time-of-flight mode. PET images were reconstructed using the iterative VuePoint FX, with 2 iterations, 24 subsets, 6.4 mm post-filter, with attenuation, scatter and decay corrections. Finally, a contrast-enhanced CT was acquired with 120 kV, 150–700 mAs Auto-mA (35 Noise Index) and 0.625 mm slice thickness. The contrast CT was performed with a split-bolus intravenous injection of iodine contrast (Omnipaque 350 mg I/ml, 0.5 g I/kg).

### FDG-PET/MRI

FDG-PET/MRI of the pelvis was performed using a 3 T PET/MRI (SIGNA PET/MRI, GE Healthcare, Milwaukee, WI) started 120 min after FDG injection. A phased-array surface coil was used in a standardized protocol consistent with the European Society of Gastrointestinal Radiology consensus guidelines [3, 22]. 40 mg of butylscopolamine bromide (Buscopan, Boehringer Ingelheim) was used as an intravenous antiperistaltic agent; if contraindicated, 1 mg (1 IE) of glucagon (Glucagon, Novo Nordisk) was used instead. No intravenous contrast agent was administered. The standard pulse sequences consisted of multiplanar 2D T2-weighted fast spin-echo (transaxial, sagittal and coronal) with 3–4 mm slice thickness, a high-resolution oblique sequence perpendicular to the rectal tumour with a slice thickness of 3 mm and a transaxial diffusion-weighted sequence (b = 200 and b = 800, including an ADC map). In addition, the protocol included a transaxial 3D T1-weighted spoiled gradient-echo sequence with 1 mm slice thickness covering the pelvic region to facilitate the identification of lymph nodes. A 30-min PET time-of-flight acquisition was acquired simultaneously using MRI. The much longer PET acquisition time than the standard clinical PET protocols, both for the CT and MRI imaging, allowed observation of PET uptake in lymph nodes which normally cannot be visualized, as shown in previous research [23]. Images were reconstructed using the iterative VuePoint FX, with 2 iterations, 24 subsets, 5.0 mm post-filter, with MRI-based attenuation correction, scatter and decay corrections. MRI parameters are summarized in Table 2.

**Table 2:** MRI protocol parameters used in the RECTOPET study

Sequence	TR [ms]	TE [ms]	ETL	FOV [mm]	Slice thickness/gap [mm]	NEX	Matrix	Acq. time	BW [Hz/px]
Sag T2 FRFSE	3900	102	20	200 × 200	3.0/0.0	3	320 × 320	04:33	223
Ax T2 FRFSE	5719	100	16	270 × 270	4.0/0.4	1	384 × 256	03:38	325
Cor T2 FRFSE	4000	102	23	220 × 220	3.0/0.0	3	320 × 320	04:16	260
T2 perp (ax)	4000	100	16	210 × 210	3.0/0.0	2	384 × 256	04:32	260
Ax DWI Focus	3500	69.4	–	240 × 120	4.0/0.0	1	160 × 80	04:47	3125
Ax T1 FSPGR	4.7	1.9	–	256 × 256	1	1	256 × 256	04:53	488

*FRFSE* Fast relaxation fast spin echo, *FSPGR* Fast spoiled gradient echo, *TR* Repetition time, *TE* Echo time, *ETL* Echo train length, *FOV* Field of view, *NEX* Number of excitations, *BW* Bandwidth, *DWI* Diffusion-weighted magnetic resonance imaging

### Evaluation of FDG-PET/CT and FDG-PET/MRI

FDG-PET/CT was evaluated according to clinical routine by radiologists, with dual certification in nuclear medicine and radiology, who had full knowledge of all the available clinical details and the standard diagnostic work-up. FDG-PET/MRI were interpreted by abdominal MRI radiologists according to clinical routine for conventional MR imaging; these personnel also had complete access to all clinical data, as well as FDG-PET/CT. The imaging results were compiled and presented at the weekly colorectal cancer multidisciplinary team meeting. Any change in diagnosis and management consequent to PET imaging reported by the clinical radiologists was noted.

### Management of the surgical specimen

**Fig. 2:**
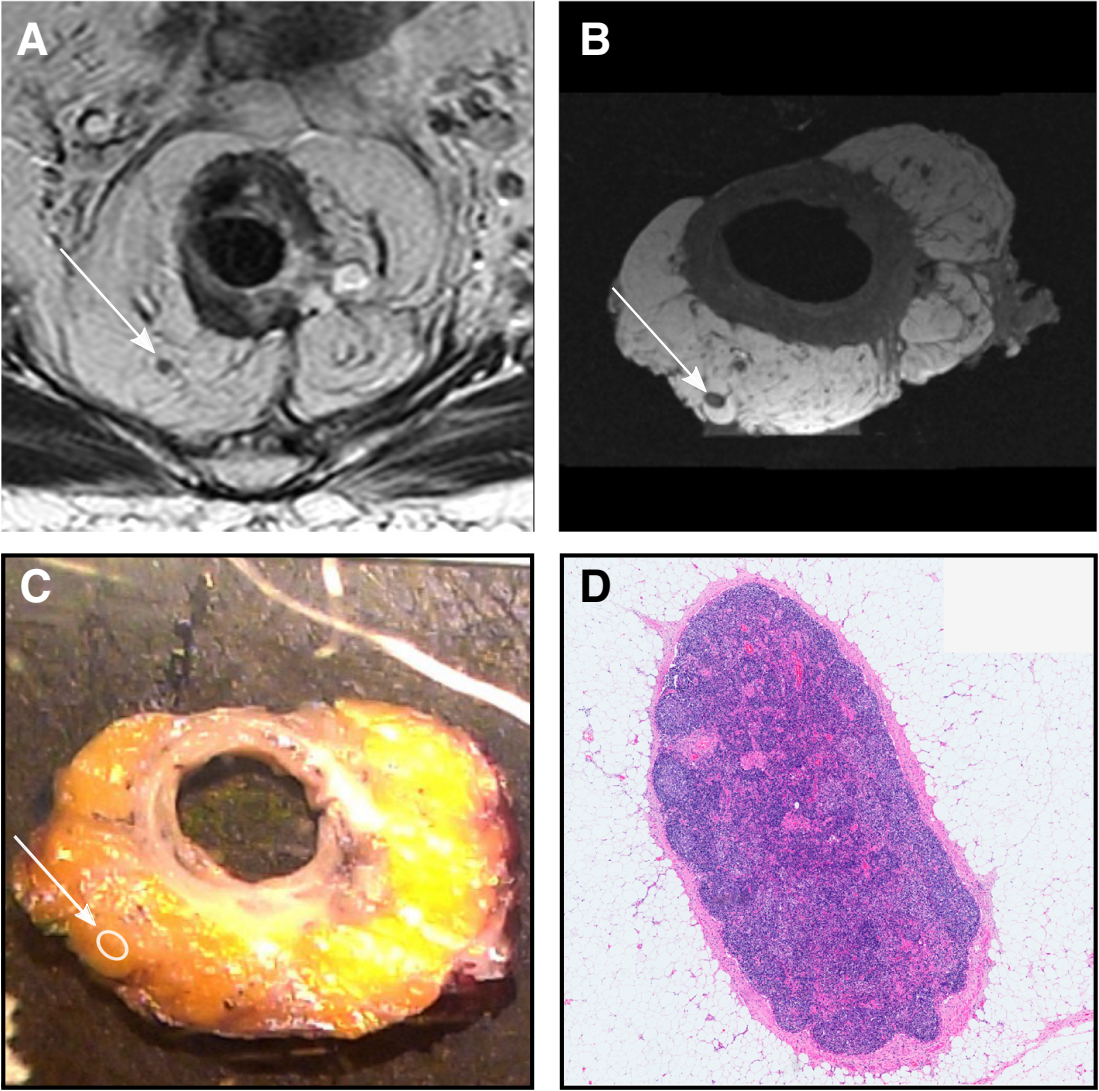
An exemple of the matching process. Anatomically matched lymph node, measuring 2.5 mm in short axis, seen in **a** transaxial T2 weighted sequence perpendicular to the tumour, **b** transaxial T1-weighted sequence MRI of the surgical specimen, **c** in the finding-by-finding description using the photographed slices arrayed numerically, and **d** at microscopy, using hematoxylin & eosin stain at 1.5x magnification, where no malignant growth was seen

In all cases where the patient underwent surgical resection, the specimen was placed in the same 3 T PET/MRI scanner used for the preoperative FDG-PET/MRI. Subsequently, anatomical matching of individual nodal structures in the radiological examinations and histopathology was performed. A detailed description and results of the matching procedure is currently a work in progress, an example of which can be seen in Fig. 2.

## Results

### Impact of FDG-PET/CT on radiological tumour stage and clinical management

**Fig. 3:**
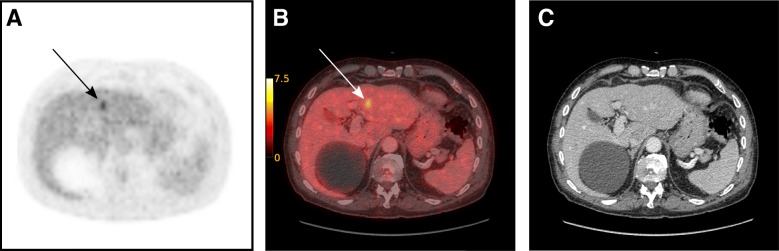
Focally increased metabolic activity in the right liver lobe without corresponding morphological changes in the second FDG-PET/CT imaging after neoadjuvant treatment. **a** FDG-PET; **b** FDG-PET/CT; **c** CT

**Fig. 4:**
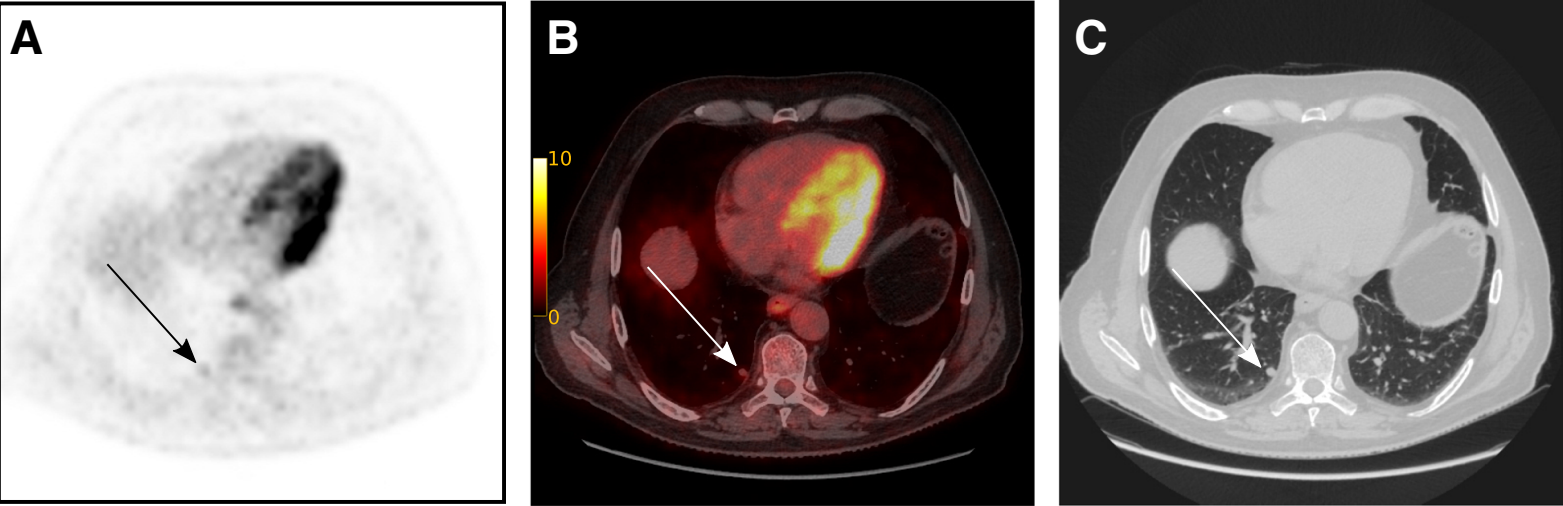
Solid subpleural nodule measuring 7 mm with increased metabolic activity (SUV max 2.8) in the right lower pulmonary lobe in the staging FDG-PET/CT. **a** FDG-PET; **b** FDG-PET/CT; **c** CT

All 24 patients included in this initial study population underwent FDG-PET/CT with a median time interval from FDG injection to examination of 60 min (interquartile range: 60–60.25 min), followed by PET/MRI with a median time interval from FDG injection to examination of 122.5 min (interquartile range: 120.75–125.25 min). Two of the patients had PET/CT and PET/MR imaging 4 and 14 days apart, respectively, due to technical problems. Of the 24 included patients, two were upstaged to M1 disease consequent to taking part in the study. For one patient, a previously non-visible lesion, with increased metabolic activity without corresponding morphological changes, was demonstrated in the right liver lobe at the second FDG-PET/CT imaging after RT (Fig. 3). MRI of the liver confirmed the diagnosis of a solitary liver metastasis, thus upstaging this patient with a cT2N0 tumour from M0 to M1. Patient management was therefore changed based on the results of FDG-PET-imaging only, and the patient underwent hepatic metastasectomy. In the other case, lung nodules were classified as malignant due to increased metabolism detected by the PET-CT (Fig. 4). Moreover, in a third case, lung nodules were evaluated as suspicious for malignancy with FDG-PET imaging based on a minor increase in metabolic activity, but were classified as indeterminate on CT and therefore as MX disease; these nodules also turned out to be unequivocally malignant on follow-up imaging with CT 6 months after surgery.

**Fig. 5:**
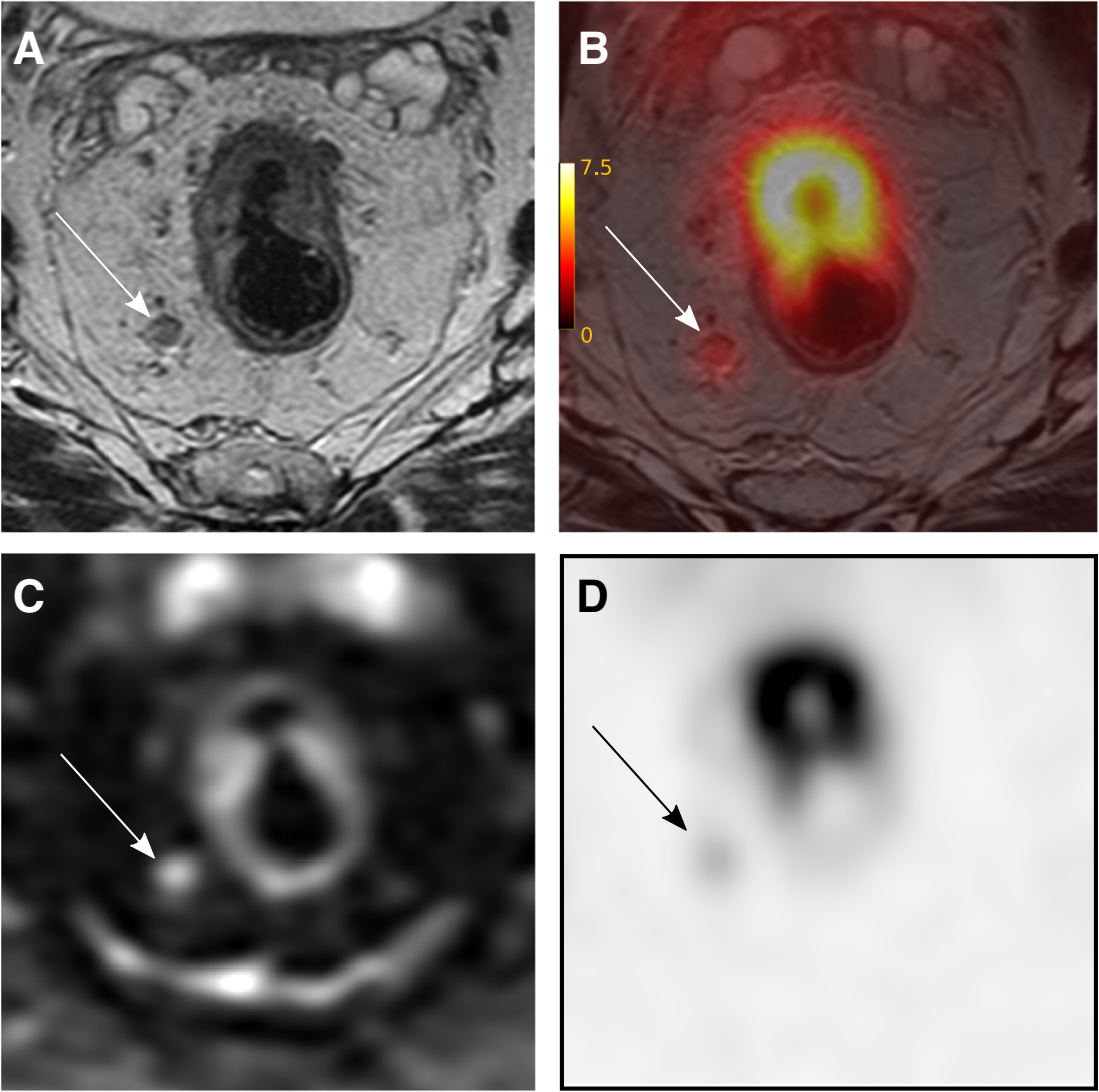
A PET/MR image of the same patient as that in Fig. 3. **a** Transaxial T2 weighted sequence perpendicular to the tumour; **b** FDG-PET/MR image with a T2 weighted MR sequence; **c** Transaxial diffusion-weighted sequence (b = 800 s/mm2) **d** Static 3D MAC PET image

Two patients had incidental findings of suspected thyroid cancers on FDG-PET imaging; one case was confirmed by histopathology (oncocytic carcinoma) after hemithyroidectomy, while the other was shown to be a benign lesion (Hürthle cell metaplasia). One patient had a finding of a duodenal polyp on FDG-PET imaging, and subsequently underwent biopsy which revealed low grade dysplasia; surveillance has been initiated. PET/MRI results were available but did not contribute to any recorded change in patient management. A typical PET/MRI image is presented in Fig. 5.

In conclusion, the clinical management of one patient was unequivocally changed in regard to rectal cancer, and resulted in a hepatic metastasectomy. One patient was upstaged to M1 disease based on the PET information from the staging FDG-PET/CT. However, this did not change the clinical management. Three more patients had findings not related to the rectal cancer; of these, two patients eventually underwent hemithyroidectomy.

## Discussion

The ongoing RECTOPET study evaluates the benefit of adding hybrid imaging to preoperative staging and restaging of rectal cancer. Although this is an observational study only, it resulted in a change in clinical management and a revised prognosis for study participants consequent to taking part. Following the standard clinical routine, PET/CT or PET/MRI would not have been performed in these cases. For the 24 included patients, liver metastasis was detected in one patient with PET/CT with subsequent surgery, and one patient had metastatic lung nodules demonstrated, though without a change in management.

There are many limitations of the current study. It is a single-centre study in a university setting, which not only reduces generalizability but also diminishes the sample size. This necessitates a relatively long study period, and the study is expected to stop recruiting patients in 2020. PET acquisition is conducted at different times and with different acquisition times for the CT and MRI hybrid scanners, which may make comparisons between modalities difficult; however, it is expected that PET/CT and PET/MR imaging will offer benefits when evaluating distant and locoregional metastases, respectively, in different anatomical regions. At a later stage a reversed sequence is planned, i.e. PET/MRI with a post-injection delay of 60 min and PET/CT at 120 min, to examine the impact of delayed imaging. It should also be noted that, in comparison to PET-CT examinations, the PET-MR imaging was performed with somewhat more post-injection period variation; the interquartile range was narrow around 120 min, while a few outliers were present. In the few instances where the examination was delayed more than 10 min in the PET/MRI scanner, this was invariably due to hardware malfunction demanding a time-consuming system reboot. Moreover, the clinical observations reported in this preliminary report must be considered provisional because the study lacked a control to establish management with and without the availability of PET/CT or PET/MRI, or the value of these techniques. Without the added metabolic information from the PET imaging, it is deemed very unlikely that the change in management reported in this study, which is based on the radiologists’ reports, would have been implemented. To properly evaluate changes in management, there must be an independent and blinded review of the examinations in the setting of a multidisciplinary team meeting assembled expressly for this purpose.

PET/CT can be used preoperatively to evaluate distant metastasis in locally advanced disease or when conventional staging is equivocal prior to surgery for liver metastases, and in patients with rising levels of carcinoembryonic antigen; nonetheless, the role of PET/CT is not completely settled [24] . Previous research has shown a higher sensitivity for PET/CT than CT alone as a single modality when assessing M stage in colorectal cancer patients before neoadjuvant therapy, with a pooled sensitivity and specificity of 91 and 95%, respectively, for PET/CT compared to a sensitivity and specificity of 91 and 16%, respectively, with CT [25]. Sensitivity and specificity for PET/CT in regard to assessment of N stage is lower, 70 and 64%, respectively [25]. However, the accuracy is highly dependent on the method of evaluation [26]. These findings might not always translate to clinical importance, but a meta-analysis of data from mixed colorectal cancer studies indicates that management changes occur in 24% of cases as a consequence of PET/CT, as reflected in the fact that extrahepatic disease is found in 32% of all cases [27]. Such benefits have not been unequivocally found in primary rectal cancer using PET/CT as part of the initial diagnostic work-up; tumour stage is typically redefined, whereas the impact on clinical management is less (ranging from 12 to 25%), and surgical plans are revised the least (0–6%) [28–30]. The added value of PET/CT for clinically important management changes seems to be greatest in more advanced tumour stages [29, 30]. In contemporary practice, some of the decade-old studies may not be entirely relevant and, because none of the above studies were population-based, they all have selection bias in common. In the current study, surgical management was unequivocally changed in one patient; this is in line with the literature and an expected preliminary result, given that hybrid imaging was routinely and not selectively used. It must also be acknowledged that lesions not related to rectal cancer were found resulting in other management changes, although with ambiguous clinical consequences, as any beneficial or adverse effects are unclear at this stage.

Quite a number of hybrid PET/MRI studies on colorectal cancer have been published in recent years, although not all concern primary staging [17–20, 23]. Paspulati et al conducted a study on 12 colorectal cancer patients, but only two had rectal cancer, for whom the authors reported an advantage of PET/MRI in T-staging. However, the primary comparison was between PET/CT and PET/MRI, and does not preclude that the reported benefit of the PET/MR imaging was due to the conventional MRI part of the PET/MRI [17]. Jeong et al studied nine patients with non-irradiated rectal cancer to evaluate metabolic activity in relation to diffusion-weighted MRI and showed an inverse correlation in the primary tumour. However, no histopathology was available, and nodal structures were not evaluated [18]. Kang et al studied a mixed colorectal cancer population and evaluated whole-body PET/MRI compared to contrast-enhanced CT. Imaging data were compared to histopathology in 12 surgical treatment-only cases, and PET/MRI was found to be superior at predicting local tumour stage; however, no comparison with diagnostic MRI was made. Moreover, three out of 19 patients in the primary staging group had management changes relating to downstaging (exclusion of metastases). It is still unclear whether conventional liver MRI and/or PET/CT would have given the same result, as no such comparison was made [19]. Plodeck et al. evaluated PET/MRI in diagnosing locoregional rectal cancer recurrence in a retrospective cohort of 44 patients, showing high accuracy; again, no comparison was made with diagnostic MRI, and primary staging was not investigated [20]. Bailey et al performed a study on 22 patients with rectal cancer, using PET/MRI in primary staging, showing that longer acquisition times improved the detection rate of radiologically abnormal and metabolically active lymph nodes; unfortunately, no histopathological comparison was performed [23].

In the current study, no additional clinical benefit of the hybrid PET/MRI was noted during the prospective clinical phase.

The RECTOPET study is ongoing. Although the benefits of adding PET/CT as well as PET/MRI to the clinical routine in this group of patients so far are admittedly small, we have shown that it is feasible to perform these examinations as well as MRI of the resected specimen within the clinical work flow. This set up would allow correlation of different morphological and functional imaging biomarkers between the hybrid imaging modalities, before and after neoadjuvant treatment, as well as a matched comparison with histopathological findings.

## Conclusion

In conclusion, replacing conventional imaging with hybrid imaging using both PET/CT and PET/MRI for staging and restaging of rectal cancer can be performed in the clinical setting. This will allow novel research on PET/MRI in particular, including a more detailed evaluation of imaging-based prognostic factors than that achieved when each examination is performed alone.

## Data Availability

The datasets used and/or analyzed during the current study are available from the corresponding author on reasonable request.

## Abbreviations

CRT: Chemoradiotherapy
CT: Computerized tomography
EMVI: Extramural venous invasion
FDG: ^18^F-fluoro-2-deoxy-D-glucose
MRF: Mesorectal fascia
MRI: Magnetic resonance imaging
PET: Positron-emission tomography
RECTOPET: REctal Cancer Trial on PET/MRI/CT
RT: Radiotherapy

## References

[1] Maas M, Lambregts DMJ, Nelemans PJ, Heijnen LA, Martens MH, Leijtens JWA (2015). Assessment of clinical complete response after Chemoradiation for rectal Cancer with digital rectal examination, endoscopy, and MRI: selection for organ-saving treatment. Ann Surg Oncol.

[2] São Julião GP, Habr-Gama A, Vailati BB, Araujo SEA, Fernandez LM, Perez RO (2017). New strategies in rectal Cancer. Surg Clin North Am.

[3] Beets-Tan Regina G. H., Lambregts Doenja M. J., Maas Monique, Bipat Shandra, Barbaro Brunella, Curvo-Semedo Luís, Fenlon Helen M., Gollub Marc J., Gourtsoyianni Sofia, Halligan Steve, Hoeffel Christine, Kim Seung Ho, Laghi Andrea, Maier Andrea, Rafaelsen Søren R., Stoker Jaap, Taylor Stuart A., Torkzad Michael R., Blomqvist Lennart (2017). Magnetic resonance imaging for clinical management of rectal cancer: Updated recommendations from the 2016 European Society of Gastrointestinal and Abdominal Radiology (ESGAR) consensus meeting. European Radiology.

[4] Beets-Tan RGH, Beets GL (2011). Local staging of rectal cancer: a review of imaging. J Magn Reson Imaging.

[5] Al-Sukhni E, Milot L, Fruitman M, Beyene J, Victor JC, Schmocker S (2012). Diagnostic accuracy of MRI for assessment of T category, lymph node metastases, and circumferential resection margin involvement in patients with rectal Cancer: a systematic review and meta-analysis. Ann Surg Oncol.

[6] Smith N. J., Barbachano Y., Norman A. R., Swift R. I., Abulafi A. M., Brown G. (2007). Prognostic significance of magnetic resonance imaging-detected extramural vascular invasion in rectal cancer. British Journal of Surgery.

[7] Chand M, Siddiqui MR, Swift I, Brown G (2016). Systematic review of prognostic importance of extramural venous invasion in rectal cancer. World J Gastroenterol.

[8] Jhaveri Kartik S., Hosseini-Nik Hooman, Thipphavong Seng, Assarzadegan Naziheh, Menezes Ravi J., Kennedy Erin D., Kirsch Richard (2016). MRI Detection of Extramural Venous Invasion in Rectal Cancer: Correlation With Histopathology Using Elastin Stain. American Journal of Roentgenology.

[9] Balyasnikova S, Haboubi N, Moran B, Brown G (2017). Histopathological and radiological reporting in rectal cancer: concepts and controversies, facts and fantasies. Tech Coloproctol.

[10] Belt EJT, Van Stijn MFM, Bril H, De Lange-De Klerk ESM, Meijer GA, Meijer S (2010). Lymph node negative colorectal cancers with isolated tumor deposits should be classified and treated as stage III. Ann Surg Oncol.

[11] Lin Q, Wei Y, Ren L, Zhong Y, Qin C, Zheng P (2015). Tumor deposit is a poor prognostic indicator in patients who underwent simultaneous resection for synchronous colorectal liver metastases. Onco Targets Ther.

[12] Kosinski L, Habr-Gama A, Ludwig K, Perez R (2012). Shifting concepts in rectal cancer management: a review of contemporary primary rectal cancer treatment strategies. CA Cancer J Clin.

[13] Evans Jessica, Patel Uday, Brown Gina (2011). Rectal Cancer: Primary Staging and Assessment After Chemoradiotherapy. Seminars in Radiation Oncology.

[14] Balyasnikova S, Brown G (2016). Optimal imaging strategies for rectal Cancer staging and ongoing management. Curr Treat Options in Oncol.

[15] Lee DH, Lee JM (2017). Whole-body PET/MRI for colorectal cancer staging: is it the way forward?. J Magn Reson Imaging.

[16] Catalano OA, Coutinho AM, Sahani DV, Vangel MG, Gee MS, Hahn PF (2017). Colorectal cancer staging: comparison of whole-body PET/CT and PET/MR. Abdom Radiol.

[17] Paspulati RM, Partovi S, Herrmann KA, Krishnamurthi S, Delaney CP, Nguyen NC (2015). Comparison of hybrid FDG PET/MRI compared with PET/CT in colorectal cancer staging and restaging: a pilot study. Abdom Imaging.

[18] Jeong JH, Cho IH, Chun KA, Kong EJ, Kwon SD, Kim JH (2016). Correlation between apparent diffusion coefficients and standardized uptake values in hybrid 18F-FDG PET/MR: preliminary results in rectal Cancer. Nucl Med Mol Imaging (2010).

[19] Kang B, Lee JM, Song YS, Woo S, Hur BY, Jeon JH (2016). Added value of integrated whole-body PET/MRI for evaluation of colorectal cancer: comparison with contrast-enhanced MDCT. Am J Roentgenol.

[20] Plodeck V, Rahbari NN, Weitz J, Radosa CG, Laniado M, Hoffmann R-T (2019). FDG-PET/MRI in patients with pelvic recurrence of rectal cancer: first clinical experiences. Eur Radiol.

[21] Boellaard R, Delgado-Bolton R, Oyen WJG, Giammarile F, Tatsch K, Eschner W (2014). FDG PET/CT: EANM procedure guidelines for tumour imaging: version 2.0. Eur J Nucl Med Mol Imaging.

[22] Taylor Fiona G. M., Swift Robert I., Blomqvist Lennart, Brown Gina (2008). A Systematic Approach to the Interpretation of Preoperative Staging MRI for Rectal Cancer. American Journal of Roentgenology.

[23] Bailey JJ, Jordan EJ, Burke C, Ohliger MA, Wang ZJ, Van Loon K (2018). Does extended PET acquisition in PET/MRI rectal Cancer staging improve results?. Am J Roentgenol.

[24] Strasberg Steven M., Dehdashti Farrokh (2010). Role of FDG-PET staging in selecting the optimum patient for hepatic resection of metastatic colorectal cancer. Journal of Surgical Oncology.

[25] Ye Y, Liu T, Lu L, Wang G, Wang M, Li J (2015). Pre-operative TNM staging of primary colorectal cancer by18F-FDG PET-CT or PET: a meta-analysis including 2283 patients. Int J Clin Exp Med.

[26] Bae SU, Won KS, Song BI, Jeong WK, Baek SK, Kim HW. Accuracy of F-18 FDG PET/CT with optimal cut-offs of maximum standardized uptake value according to size for diagnosis of regional lymph node metastasis in patients with rectal cancer. Cancer Imaging. 2018;18:18–32. 10.1186/s40644-018-0165-5 PMC6137872 30217167

[27] Herrmann K, Lopci E, Rubello D, Maffione AM, Giammarile F, Bluemel C (2014). Diagnostic accuracy and impact on management of 18F-FDG PET and PET/CT in colorectal liver metastasis: a meta-analysis and systematic review. Eur J Nucl Med Mol Imaging.

[28] Davey K, Heriot AG, Mackay J, Drummond E, Hogg A, Ngan S (2008). The impact of 18-fluorodeoxyglucose positron emission tomography-computed tomography on the staging and management of primary rectal cancer. Dis Colon Rectum.

[29] Ozis SE, Soydal C, Akyol C, Can N, Kucuk ON, Yagcı C (2014). The role of 18F-fluorodeoxyglucose positron emission tomography/computed tomography in the primary staging of rectal cancer. World J Surg Oncol.

[30] Eglinton T, Luck A, Bartholomeusz D, Varghese R, Lawrence M (2009). Positron-emission tomography/computed tomography (PET/CT) in the initial staging of primary rectal cancer. Color Dis.

